# Competing with greater impairment: age distribution and competitive outcomes in Paralympic athletes with high support needs

**DOI:** 10.3389/fspor.2026.1872208

**Published:** 2026-07-15

**Authors:** Rafael Lima Kons, Luiz Gustavo T. Santos, Gennaro Apollaro, Marilia Passos Magno e Silva, João Paulo Casteleti de Souza, María Isabel Cornejo, Lorenzo Rum, Matías Henríquez

**Affiliations:** 1 Department of Physical Education, Faculty of Education, Federal University of Bahia, Bahia, Brazil; 2 Sports Development Department, Brazilian Paralympic Committee, São Paulo, Brazil; 3 Department of Neuroscience, Rehabilitation, Ophthalmology, Genetics and Maternal Child Health, University of Genoa, Genoa, Italy; 4 Laboratory of Adapted Physical Activity, Institute of Health Sciences, Postgraduate Program in Human Movement Sciences, Federal University of Pará, Belém, Brazil; 5 Escuela de Kinesiología, Facultad de Salud, Universidad Santo Tomas, Santiago, Chile; 6 Magíster en Ciencias de la Actividad Física y del Deporte Aplicadas al Entrenamiento, Rehabilitación y Reintegro Deportivo, Universidad Santo Tomas, Santiago, Chile; 7 Department of Biomedical Sciences, University of Sassari, Sassari, Italy

**Keywords:** athlete development, classification system, developmental pathways, impairment severity, para-sport, performance trajectory

## Abstract

This study examined the age distribution of Paralympic athletes with high support needs at the Paris 2024 Paralympic Games, analyzing differences according to sex, competitive achievement, and discipline. A total of 1,263 athletes from eight individual sports were included. Between-group comparisons were conducted using the Mann–Whitney *U* and the Kruskal–Wallis test with Bonferroni *post hoc*, while Chi-square tests assessed associations between age categories at the 5% level. Male athletes had a higher median age than female athletes (33 vs. 29 years; *p* < 0.001), whereas no difference was observed between medalists and non-medalists (*p* = 0.62). Significant variation in age was observed across disciplines (*p* < 0.001), with Para archery (40 years), Para table tennis (42 years), and Para equestrian (37 years) showing the highest median ages, while Para swimming (26 years) had the youngest median age. Age category analysis indicated a predominance of athletes aged 30 or older, particularly among males and in specific disciplines. These findings suggest that age patterns among athletes with high support needs are influenced by sex and sport-specific context rather than competitive achievement, highlighting the importance of considering classification level when examining long-term performance trajectories in Paralympic sport.

## Introduction

Elite performance and athlete career pathways in Paralympic sport are the outcomes of a multifaceted developmental process, in which long-term progression is shaped by the interaction of individual and contextual factors (1). Rather than resulting from isolated variables, elite performance and the development of para-athletes reflect the complex interaction between individual characteristics, environmental conditions, and task demands, including access to training infrastructure, coaching expertise, social and institutional support systems, and impairment-related factors (1–3). These elements are embedded within structured para-sport development pathways that guide progression through the stages of participation (2). Within this framework, para-athletes' training backgrounds, age at entry, specialization, impairment-related characteristics, and opportunities for long-term, systematic preparation play a critical role in shaping trajectories toward sustained elite achievement (1, 3–5).

Given this multifactorial landscape, the way para-athletes are grouped and regulated within competitive structures can substantially influence both their developmental opportunities and competitive performance (1, 3–5). Within the broader context of functioning in people with disabilities, classification plays a key role in Paralympic sport, as sport-specific systems define eligibility and group para-athletes according to the impact of their impairment on performance, aiming to minimize its influence on competition outcomes and ensure that success is primarily determined by sporting skill (6). Each para-sport classification system establishes minimum impairment criteria, which serve as the threshold for eligibility to compete rather than as a mechanism to reduce the impact of impairment. Beyond this threshold, athletes may present varying levels of impairment severity, leading to greater activity limitation and forming a continuum across sport classes, particularly among those with higher support needs (6–8). In the present study, “athletes with high support needs” is used as an operational umbrella term rather than as a formal IPC classification category. It refers to athletes competing in selected sport classes that, within each sport-specific classification system, are associated with greater activity limitation and/or the need for sport-specific support, including guides, pilots, handlers, personal assistants, or highly adapted equipment, when applicable. Depending on the discipline and governing body, additional divisions such as sex, age, and weight categories may further structure competition (9). Beyond these formal mechanisms, emerging evidence suggests that age distribution may be an influential contextual factor in para-athlete development, with potential implications for long-term performance and success at the highest competitive levels (10–12).

Recent evidence suggests that age distribution may be relevant in Paralympic sport. Kons et al. (13), analyzing 5,540 athletes from the Paris 2024 Paralympic Games across 13 disciplines, reported variation between sports, with relatively older profiles in Para equestrian, Para archery, and Para powerlifting, and younger profiles in Para swimming, Para taekwondo, and Para athletics. Males exhibited a slightly higher median age than females (30 vs. 28 years), and no meaningful differences were observed between medalists and non-medalists (13). Nevertheless, research across multiple disciplines, including Para canoeing (14), Para swimming (15–17), Wheelchair racing (18), Wheelchair rugby (19), Para powerlifting (20, 21), and Para judo (13) demonstrates distinct age profiles and performance trajectories within sports. Overall, these findings indicate that age, although not a direct predictor of medal attainment, may be a relevant factor in athlete development and long-term performance in Paralympic sport.

Building on this perspective, it is relevant to consider how classification structures intersect with age-related patterns in Paralympic sport (13), as the relationship between sport-specific classification structures and age distribution among Paralympic athletes remains largely unexplored in the current literature, particularly among athletes with high support needs. Para athletes with high support needs, as operationally defined in this study, have greater activity limitations and/or require additional sport-specific support depending on the discipline, and may therefore experience distinct developmental trajectories, competitive exposures, and career dynamics compared to those with lower impairment severity or lower sport-specific support requirements (1). Previous research suggests that these athletes are underrepresented in para-sports (22) and face greater barriers to participation, including higher impairment severity, the need for specialized equipment, adapted training environments, and increased reliance on multidisciplinary support (23, 24). As classification structures shape training demands (3–5), competitive opportunities, and progression pathways (1, 5), age-related patterns in athletes with high support needs may differ from those observed in the broader Paralympic population. Therefore, examining age distribution in this subgroup is scientifically relevant for understanding Para athlete development and practically relevant for informing talent identification, long-term athlete development, competition planning, and the provision of appropriate support systems.

Considering the broader Paralympic context and the characteristics of athletes with high support needs within classification systems, this study aimed to examine age distribution across multiple sports, including differences by competitive outcomes, sex, and discipline. Describing these patterns may help inform talent development pathways in Paralympic sport (1, 5), support evidence-based planning of training and competition structures (3–5), and contribute to more inclusive and context-sensitive sport systems for athletes with high support needs.

## Methods

### Study design

This descriptive, exploratory, cross-sectional study examined age-related performance data from eight individual para-sports at the Paris 2024 Paralympic Games, focusing on athletes with high support needs, defined as those competing in sport classes with the greatest activity limitation due to impairment severity (25). As the study includes only Paralympic athletes competing at the highest level, the sample was classified as Tier 5: World Class according to the participant classification framework (26).

### Data collection and procedures

Data were extracted from the Official Results Books from the public official website (https://www.ipc-services.org/hira/results-books), which include information on each athlete's name, sex, birth date, sport class, and competitive outcomes (e.g., final ranking or medal status). Archived databases from open-access websites have been used in similar investigations, and no ethical issues arise from their analysis, as the data are publicly available and obtained secondarily rather than generated experimentally (27). This approach to data utilization aligns with the ethical principles outlined in the Belmont Report (27), ensuring that participants' autonomy and confidentiality are upheld. The dataset analysis included information from multiple variables: Eight individuals Paralympic sports (Para archery, Para athletics, Para cycling, Para equestrian, Para judo, Para swimming, Para table tennis, and Para triathlon), sex (male and female), and competitive achievement (medalists and non-medalists). In addition, age distributions were categorized into four groups, based on prior research (13): under 20 years, 20–25 years, 25–30 years, and over 30 years. The age was calculated using the formula: (competition date − birth date)/365.25 (19). The official start date of the Paris 2024 Paralympic Games (i.e., the opening ceremony) was used as the reference date for all calculations. The determination of age was based on calculating the age of athletes competing in a pinnacle event such as the Paris 2024 Paralympic Games (28).

### High support needs athletes classifications

All athletes had an official sport class assigned according to the sport-specific Paralympic classification system for each para-sport, which aims to minimize the impact of eligible impairments on competition outcomes by grouping athletes according to the extent to which impairments affect sport performance (6, 22). In this study, “high support needs” was used as an operational umbrella term to refer to selected sport classes within each sport-specific classification system that include athletes with greater sport-related activity limitations and/or substantial competition-related support requirements. Because classification systems differ across Paralympic sports, this term was not treated as a single formal classification category. Instead, it was applied to identify classes in which athletes commonly present a greater functional impact of impairment on sport performance or require sport-specific support, such as guides, pilots, handlers, assistants, or highly adapted equipment, when applicable. The selected classes were defined *a priori* based on the International Paralympic Committee Athlete Classification Code and the respective sport-specific classification rules, and generally corresponded to classes associated with more severe impairments and greater support needs for performance (8, 29). In some cases, athletes in these classes may also be eligible for additional support personnel, which, although essential for equitable participation, can increase logistical demands and affect the organization of certain events (3). Table 1 presents the analytical dataset, which comprised 1,263 Paralympic athletes across eight sports.

**Table 1:** Summary of statistical tests used in the study.

Analysis	Variables	Statistical test	*Post hoc* test	Effect size	Significance level
Normality assessment	Age and other continuous variables	Kolmogorov–Smirnov test	Not applicable	Not applicable	*p* < 0.05
Sex comparison	Age by sex	Mann–Whitney *U* test	Not applicable	*r* = *Z*/√*N*	*p* < 0.05
Competitive achievement comparison	Age by medal status	Mann–Whitney *U* test	Not applicable	*r* = *Z*/√*N*	*p* < 0.05
Discipline comparison	Age across eight Paralympic disciplines	Kruskal–Wallis test	Pairwise Mann–Whitney *U* tests	*ε*^2^ for omnibus test; *r* = *Z*/√*N* for pairwise comparisons	*p* < 0.05 for omnibus test; *p* ≤ 0.002 for Bonferroni-adjusted pairwise comparisons
Association analyses	Sex, competitive achievement, and discipline with age categories	Chi-square test of independence	Not applicable	Cramer's *V*	*p* < 0.05

### Statistical analysis

The Kolmogorov–Smirnov test indicated that all considered variables were non-normal. Therefore, descriptive data are presented as median (interquartile range) (minimum–maximum). First, the Mann–Whitney *U* test was performed to detect differences in age by sex (female and male) and competitive ranking (medalist and non-medalist). For these comparisons, the effect size *r* was reported for each *p*-value. Second, the Kruskal–Wallis test followed by the Mann–Whitney *U post hoc* test was used to detect differences in athletes' ages across Paralympic disciplines (i.e., Para archery, Para athletics, Para cycling, Para equestrian, Para judo, Para swimming, Para table tennis, Para triathlon). For the Kruskal–Wallis omnibus test, effect size was reported using epsilon-squared (*ε*^2^), with values interpreted as small (0.01–<0.06), moderate (0.06–<0.14), and large (≥0.14). Considering the 28 pairwise comparisons conducted across the eight Paralympic disciplines, the alpha level was adjusted using a Bonferroni correction (0.05/28 = 0.0018; rounded to *p* < 0.002) to reduce the risk of inflated Type I error. For all these analyses, effect size *r* was calculated by the formula: *r* = *Z*/√*N*; and the magnitudes of the effect size were interpreted with values of ≤0.09 = *trivial*, 0.10–0.29 = *small*, 0.30–0.49 = *medium*, and ≥0.50 = *large* (30). Finally, chi-square (*χ*^2^) tests of independence were performed to identify the association of sex, competitive achievement, and Paralympic discipline with the age categories (i.e., <20, 20–25, 25–30, >30). For these analyses, effect size was reported using Cramer's *V*, and magnitudes were interpreted as small (0.06–0.17), medium (0.18–0.29), or large (≥0.30) (31). Statistical significance was defined as *p* < 0.05, except for Bonferroni-adjusted pairwise *post hoc* comparisons, and all analyses were performed using IBM SPSS Statistics for Windows, version 25.0 (IBM Corp., Armonk, NY, USA).

## Results

Table 2 presents a comparison of age distribution by sex, competitive achievement, and discipline at the 2024 Paris Paralympic Games. There was a significant difference in age between male and female Paralympic athletes (*U* = 161,702.00; *p* < 0.001; *r* = 0.16, small), with male athletes being older than female athletes. In contrast, no significant difference was observed between medalists and non-medalists (*U* = 162,019.50; *p* = 0.620; *r* = 0.01, trivial). A significant effect of discipline was also found (*χ*^2^_(7)_ = 54.882, *p* < 0.001; *ε*^2^ = 0.04, small), indicating variability in age distribution across sports. *post-hoc* analyses showed that athletes in Para archery were older than athletes in Para athletics (*U* = 2,450.50; *p* < 0.001; *r* = 0.19, small), Para judo (*U* = 506.00; *p* < 0.001; *r* = 0.34, medium), and Para swimming (*U* = 1,542.00; *p* < 0.001; *r* = 0.27, small). Para athletics athletes were younger than those in Para equestrian (*U* = 12,425.50; *p* < 0.001; *r* = 0.19, small) and Para table tennis (*U* = 4,611.50; *p* = 0.002; *r* = 0.16, small), but older than Para swimming athletes (*U* = 53,598.00; *p* < 0.001; *r* = 0.31, medium). Para cycling athletes were older than Para swimming athletes (*U* = 20,890.50; *p* < 0.001; *r* = 0.39, medium). Similarly, Para equestrian athletes were older than Para swimming athletes (*U* = 8,894.00; *p* < 0.001; *r* = 0.36, medium), as did Para judo athletes (*U* = 10,042.00; *p* < 0.001; *r* = 0.26, small). Finally, Para swimming athletes were younger than Para table tennis (*U* = 3,674.00; *p* < 0.001; *r* = 0.25, small) and Para triathlon athletes (*U* = 2,062.00; *p* < 0.001; *r* = 0.27, small).

**Table 2:** Pairwise *post hoc* comparisons of athlete age across Paralympic disciplines.

Comparison	*U*	*p*-value	*r*	Effect size magnitude	Significant after Bonferroni correction
Para archery vs. Para athletics	2,450.50	<0.001	0.19	Small	Yes
Para archery vs. Para cycling	1,486.00	0.006	0.19	Small	No
Para archery vs. Para equestrian	901.50	0.189	0.12	Small	No
Para archery vs. Para judo	506.00	<0.001	0.34	Medium	Yes
Para archery vs. Para swimming	1,542.00	<0.001	0.27	Small	Yes
Para archery vs. Para table tennis	410.00	0.740	0.04	Trivial	No
Para archery vs. Para triathlon	241.50	0.082	0.24	Small	No
Para athletics vs. Para cycling	29,829.50	0.003	0.13	Small	No
Para athletics vs. Para equestrian	12,425.50	<0.001	0.19	Small	Yes
Para athletics vs. Para judo	13,988.00	0.436	0.04	Trivial	No
Para athletics vs. Para swimming	53,598.00	<0.001	0.31	Medium	Yes
Para athletics vs. Para table tennis	4,611.50	0.002	0.16	Small	Yes
Para athletics vs. Para triathlon	3,503.00	0.003	0.15	Small	No
Para cycling vs. Para equestrian	7,320.50	0.051	0.12	Small	No
Para cycling vs. Para judo	6,546.00	0.126	0.09	Trivial	No
Para cycling vs. Para swimming	20,890.50	<0.001	0.39	Medium	Yes
Para cycling vs. Para table tennis	2,562.00	0.021	0.15	Small	No
Para cycling vs. Para triathlon	2,192.00	0.153	0.10	Small	No
Para equestrian vs. Para judo	2,722.50	0.006	0.21	Small	No
Para equestrian vs. Para swimming	8,894.00	<0.001	0.36	Medium	Yes
Para equestrian vs. Para table tennis	1,564.00	0.692	0.04	Trivial	No
Para equestrian vs. Para triathlon	1,272.50	0.992	0.01	Trivial	No
Para judo vs. Para swimming	10,042.00	<0.001	0.26	Small	Yes
Para judo vs. Para table tennis	934.00	0.003	0.27	Small	No
Para judo vs. Para triathlon	764.00	0.015	0.23	Small	No
Para swimming vs. Para table tennis	3,674.00	<0.001	0.25	Small	Yes
Para swimming vs. Para triathlon	2,062.00	<0.001	0.27	Small	Yes
Para table tennis vs. Para triathlon	402.50	0.169	0.17	Small	No

Pairwise *post hoc* comparisons between disciplines were performed using Mann–Whitney *U* tests with Bonferroni correction. The Bonferroni-adjusted significance level was set at *p* < 0.002. Effect size was calculated as *r* = *Z*/√*N* and interpreted as trivial (≤0.09), small (0.10–0.29), medium (0.30–0.49), and large (≥0.50).

**Figure 1:**
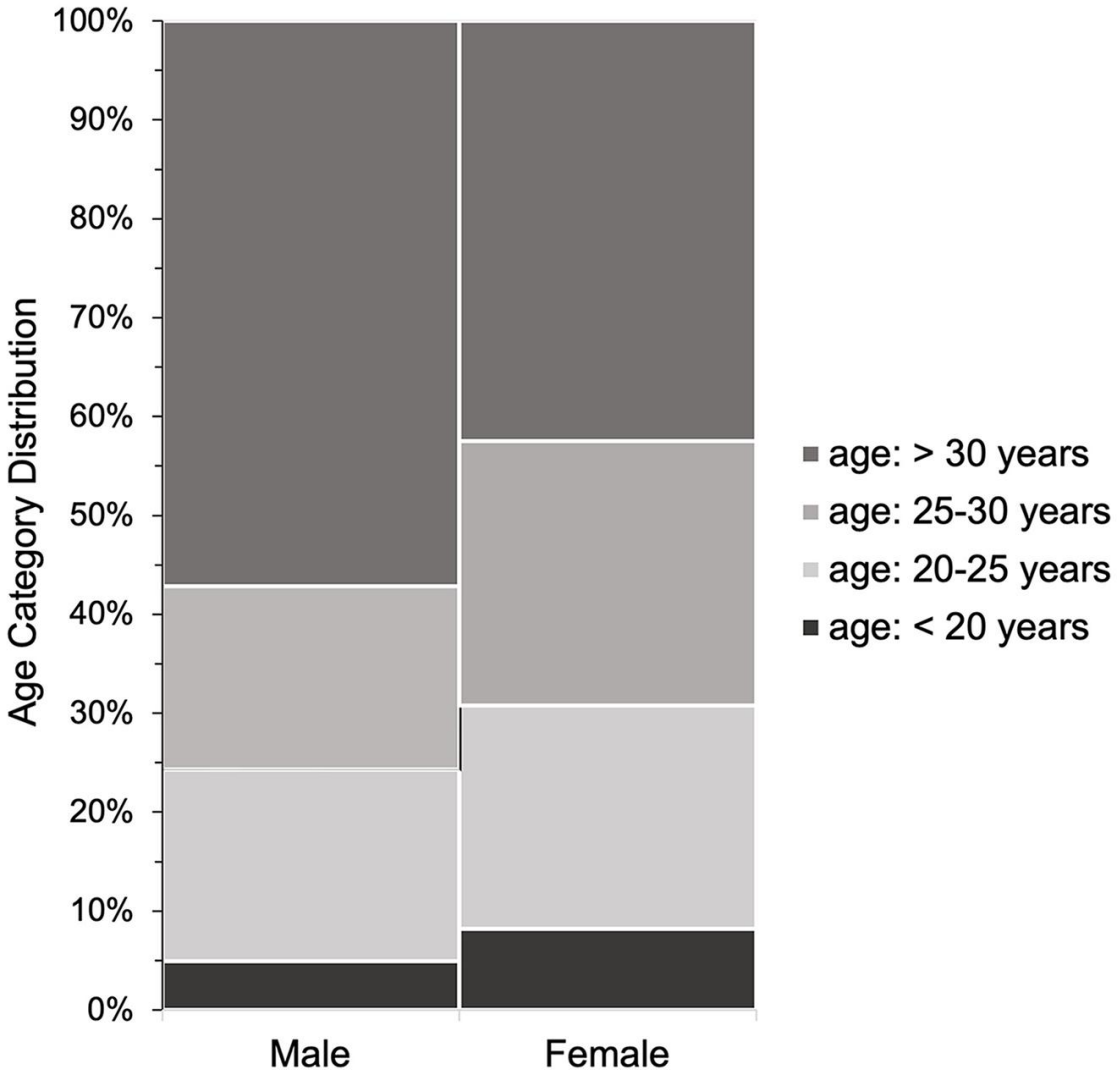
Age category distribution of male and female athletes who participated in the Paris 2024 Paralympic Games. The stacked bar chart displays the proportion of athletes (eligibles) in four age categories: <20 years, 20–25 years, 25–30 years, and >30 years.

**Figure 2:**
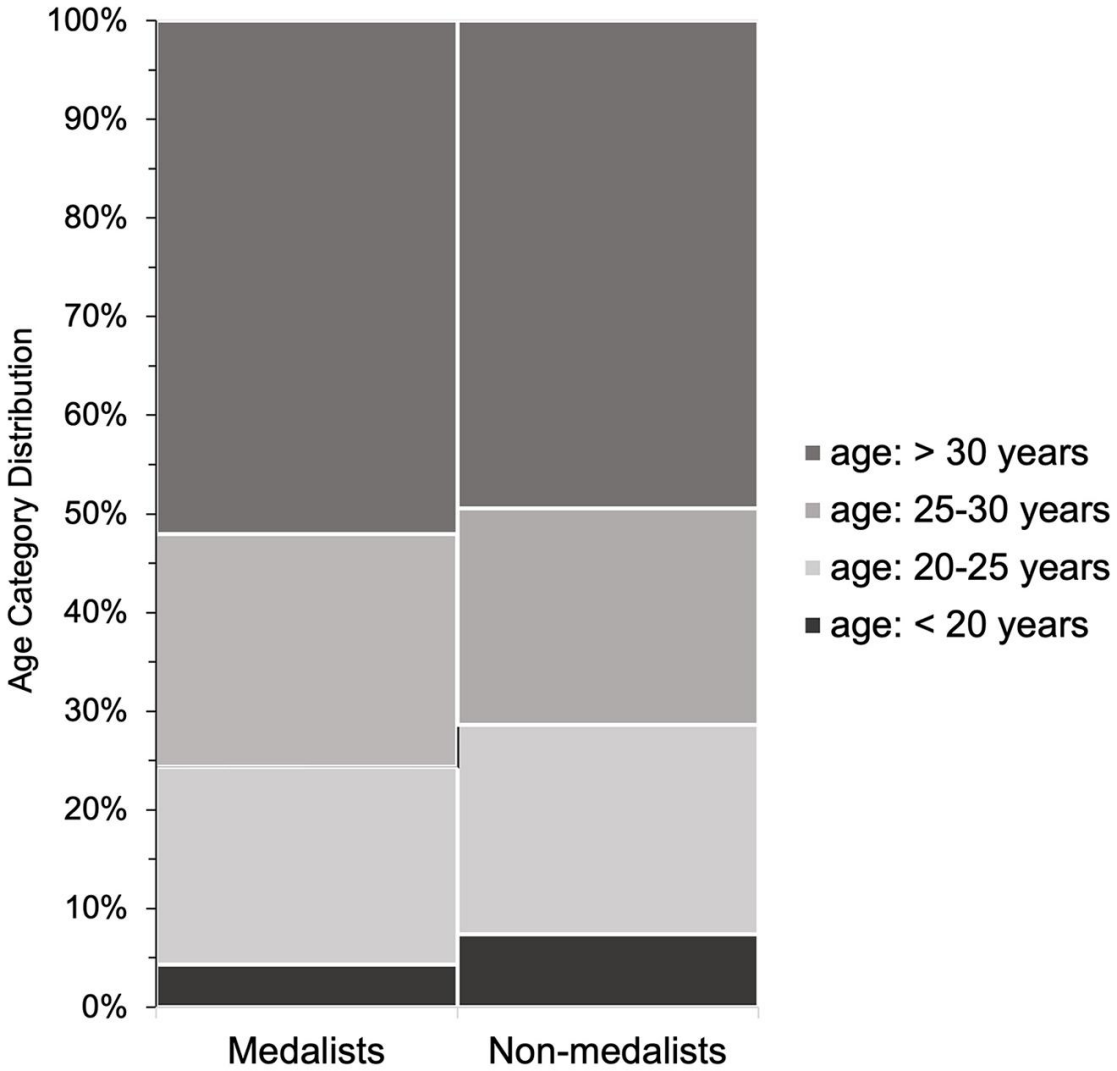
Age category distribution of medalists and non-medalists athletes who participated at the Paris 2024 Paralympic Games. The stacked bar chart displays the proportion of athletes in four age categories: <20 years, 20–25 years, 25–30 years, and >30 years.

**Figure 3:**
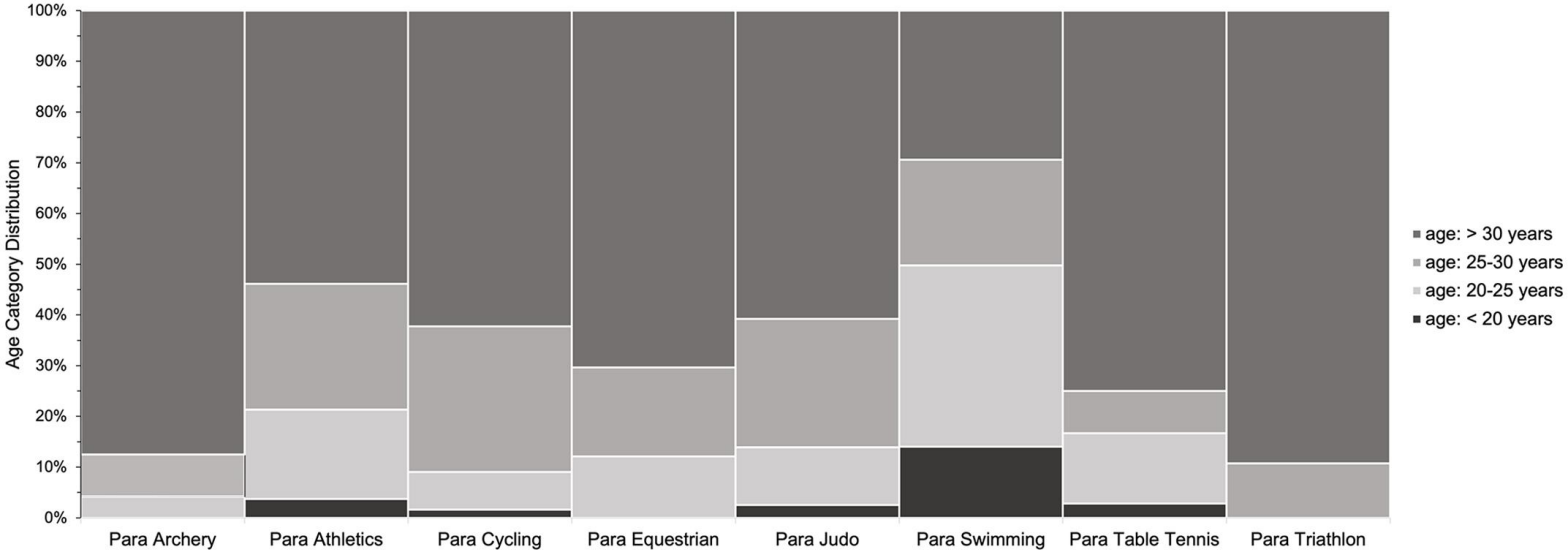
Age category distribution of athletes from different disciplines who participated in the Paris 2024 Paralympic Games. The stacked bar chart displays the proportion of athletes in four age categories: <20 years, 20–25 years, 25–30 years, and >30 years.

Figures 1–3 present the associations of the sex (i.e., female and male), competitive achievement (i.e., medalist and non-medalist), and paralympic discipline (i.e., Para archery, Para athletics, Para cycling, Para equestrian, Para judo, Para swimming, Para table tennis, and Para triathlon), with the age categories (i.e., <20, 20–25, 25–30, >30), respectively. Significant associations were found for sex (*χ*^2^_(3)_ = 29.666; *p* < 0.001; *V* = 0.153, *small*) and Paralympic sport (*χ*^2^_(21)_ = 229.725; *p* < 0.001; *V* = 0.246, *medium*), indicating a higher distribution of athletes in the group >30 years age group compared to the other age groups. On the contrary, no significant association emerged for competitive achievement (*χ*^2^_(3)_ = 4.581; *p* = 0.205; *V* = 0.060, *small*).

## Discussion

The present study investigated the age distribution of Paralympic athletes with high support needs competing at the Paris 2024 Paralympic Games, examining differences according to competitive achievement, sex, and discipline. Overall, the findings indicate that age patterns are not uniform but vary by sex and sport-specific context. The male groups presented higher median ages than females, while considerable variability was observed across disciplines. Notably, athletes with high support needs demonstrated relatively elevated age profiles, with median ages ranging from 26 to 42 years and overall age spans from 13 to 69 years. In addition, traditionally structured sports tended to display more consistent age profiles, whereas marked differences emerged between disciplines such as Para archery, Para table tennis, and Para swimming. Although these findings describe differences in age distribution across sex and disciplines, the mechanisms underlying these patterns cannot be directly determined from the present cross-sectional dataset. Therefore, the explanations discussed below should be interpreted as plausible interpretations supported by previous literature rather than causal conclusions derived from the current data. Taken together, these findings reinforce the notion that performance trajectories in Paralympic sport are shaped by the interaction between classification systems, sport-specific demands, and the organization of development pathways (5, 29). In addition, delayed access to sports opportunities and lower participation rates, common in this population, may influence these age patterns across sports (6).

Although most athletes were over 30 years of age in both sexes, a distinct pattern emerged in the distribution of female athletes, with a higher proportion of individuals under 20 years competing in high-support needs classes than males. This may partly reflect recent efforts to expand female participation in paralympic sport (32). Initiatives promoted by the International Paralympic Committee have emphasized increasing access for girls and women, particularly at early stages of sport development (32). In this context, the greater representation of younger female athletes may indicate an early impact of policies aimed at reducing sociocultural barriers and strengthening talent identification pathways (4). This finding may indicate early achievement of performance milestones, particularly among para-athletes with congenital impairment (3), representing a pattern that contrasts with the commonly assumed trend of later specialization in this population (26). Notably, no meaningful differences in age distribution were observed between medalists and non-medalists, suggesting that, within high-support-needs groups, competitive success may be less strongly associated with age-related factors and more influenced by structural, developmental, and contextual factors (13). Rather than reflecting only performance-related factors, these findings may also indicate broader shifts in participation opportunities and structural support for women with higher levels of impairment in Paralympic sport (32, 33).

Within this context, comparisons across disciplines provide further insight into how these structural and functional elements influence athlete development (1). Paralympic sports are characterized by distinct physiological, technical, and organizational demands (13), which contribute to different timelines for progression and peak performance (1), particularly among athletes with high support needs, as suggested by the present findings. Among the disciplines analyzed, Para swimming showed the greatest age distribution variability. This can be explained by the fact that this modality integrates athletes with physical, visual, and intellectual impairments within a single competitive structure, potentially increasing heterogeneity in developmental trajectories (15–17). Moreover, this variability may reflect two interrelated factors. First, evidence suggests that the Para swimming classification system does not consistently differentiate performance between adjacent classes (34). Second, athletes with high support needs exhibit heterogeneous functional characteristics and training responses (23). As a result, athletes with diverse profiles may perform at similar levels despite belonging to different classes. Although Para athletics similarly encompasses these impairment groups, it showed higher age values than swimming, suggesting that athletes in athletics may require longer periods to reach elite performance or remain competitive for longer, considering acquired and congenital impairments (1). These differences align with evidence indicating that peak performance in Para athletics varies by event, impairment type, and sex, with broad age ranges among top performers, and may be further influenced by impairment origin and sport-specific adaptation processes (18). In contrast, the younger age profile observed in swimming, particularly among athletes under 20 years of age, may reflect differences in recruitment systems, earlier entry into structured training environments, or shorter pathways to high-level performance (1, 5).

These sport-specific patterns become even more evident when considering the interaction between impairment characteristics and each sport's performance demand (8). The differences in age between Para archery and disciplines such as Para athletics, Para judo, and Para swimming may be partially explained by the nature of eligible impairments and the physiological and technical demands of each sport. This perspective aligns with recent findings from studies examining broader Paralympic classification groups, which have identified comparable age-related patterns across disciplines (13). While Para judo is restricted to athletes with visual impairments and involves high-intensity, intermittent efforts (35), Para archery is practiced by athletes with physical impairments and relies more on technical precision and fine motor control (36). These differences may be particularly relevant for athletes with high support needs, for whom the interaction between impairment severity and sport-specific demands can further shape performance trajectories and career longevity (4, 5, 29). In this context, Para archery may enable greater sustainability in high-level performance, as its technical nature may mitigate the impact of physical limitations associated with higher support needs and enable the use of assistive devices that support competitive participation among athletes with more severe impairments (37). Accordingly, pathways involving transitions between sports emerge as a relevant factor (1, 4), as technically oriented disciplines such as archery may offer experienced athletes opportunities to extend their competitive careers.

Some limitations should be considered when interpreting the present findings. First, not all Paralympic sports involving athletes with high support needs were included, particularly Para rowing and boccia, which may limit the generalizability of the results. Additionally, the lack of stratification by impairment type and origin within each sport class limits the ability to capture intra-class heterogeneity and to disentangle potential compositional effects associated with classification systems (29). The sex × discipline interaction was not formally tested because several sex-by-discipline subgroups were highly unbalanced or had very small sample sizes, which could compromise the reliability of interaction estimates. Therefore, sex and discipline were analyzed separately, and future studies with larger and more balanced samples should examine whether sex-related age differences are discipline-specific. Furthermore, the cross-sectional design limits causal inference relationships and the analysis of changes over time. The use of competition-based data includes only Paralympic-level athletes, potentially introducing selection bias and limiting the representation of broader participation pathways. In addition, key contextual factors such as training history, age of sport entry, impairment onset (congenital vs. acquired), and access to support systems were not considered, despite their potential influence on performance trajectories, particularly among athletes with high support needs. Given the complexity of Paralympic sport structures, future research should incorporate longitudinal approaches and more detailed analyses of classification and impairment levels, alongside contextual and developmental variables, to further refine the understanding of athlete development and performance patterns.

## Conclusion

The present study provides novel insights into the age distribution of Paralympic athletes with high support needs, suggesting that age-related patterns are primarily shaped by sex and sport-specific context rather than competitive achievement. Overall, athletes in this group presented relatively elevated age profiles, with a predominance of individuals over 30 years of age, suggesting extended performance trajectories even among those with greater functional limitations. These findings reinforce the importance of considering classification level when examining athlete development and performance in Paralympic sport. From an applied perspective, the results support the need for context-specific talent development strategies that account for both sport demands and the characteristics of athletes with high support needs.

## Data Availability

Publicly available datasets were analyzed in this study. This data can be found here: https://www.ipc-services.org/hira/results-books.
